# Helicopter emergency medical service (HEMS) activity after increased distance to out-of-hours services: an observational study from Norway

**DOI:** 10.1186/s12873-020-00377-0

**Published:** 2020-11-02

**Authors:** Dag Ståle Nystøyl, Jo Røislien, Øyvind Østerås, Steinar Hunskaar, Hans Johan Breidablik, Erik Zakariassen

**Affiliations:** 1 Department of Research, Norwegian Air Ambulance Foundation, Oslo, Norway; 2 Health Services Research Group, Department of Global Public Health and Primary Care, University of Bergen, PBox 7810, 5020 Bergen, Norway; 3 Faculty of Health Sciences, University of Stavanger, Stavanger, Norway; 4 Department of Anaesthesia and Intensive Care, Haukeland University Hospital, Bergen, Norway; 5 National Centre for Emergency Primary Health Care, NORCE Norwegian Research Centre, Bergen, Norway; 6 Centre of Health Research, Førde Hospital Trust, Førde, Norway

**Keywords:** Emergency medical services, Primary health care, Air ambulances, Norway, HEMS, General practitioners, After-hours care, Out-of-hours medical care

## Abstract

**Background:**

Organizational changes in out-of-hour (OOH) services may have unintended consequences for other prehospital services. Reports indicate an increased use of helicopter emergency medical services (HEMS) after changes in OOH services in Norway due to greater geographical distances for the on-call doctors. We investigated whether HEMS dispatches increased when nine municipalities in Sogn og Fjordane County merged into one large inter-municipal OOH district.

**Methods:**

All primary dispatches of the HEMS in the county between 2004 and 2013 were included. We applied interrupted time series regression to monthly aggregated data to evaluate the impact of the organizational change 1 April 2009. The nine target municipalities were compared to the rest of the municipalities in the county, which served as a control group. A quasipoisson model adjusted for seasonality was found to be most applicable.

**Results:**

We included 8,751 dispatches, 5,009 (57.2%) of which were completed with a patient encounter. Overall, we found no alteration in requests for HEMS after 2009 (*p* = 0.251). Separate analyses of the target municipalities and control group revealed no significant increase after 2009 (*p* = 0.400 and *p* = 0.056, respectively). When categorizing the municipalities into urban or rural, we found a general increase in HEMS dispatches for the rural group over the 10-year span (*p* = 0.045) but no added increase after 2009 (*p* = 0.502). The urban subgroup showed no change. Distance from the OOH service in regards to travel increased within the nine municipalities after 2009, median [quartiles] (5.0[3.0, 6.2] km vs 26.5[5.0, 62.2] km, *p* < 0.001).

**Conclusion:**

After relocating nine local OOH services into one large inter-municipal OOH district, we found no increase in requests for HEMS.

## Background

The Helicopter Emergency Medical Service (HEMS) is part of the prehospital emergency medical service in many industrialized countries [1]. Studies have shown varying effects on health of such services [2], but their use is increasing worldwide [3]. In Norway, HEMS is an integrated part of the public prehospital emergency medical services, together with ground ambulances and primary care out-of-hours (OOH) services [4]. Ambulances and OOH services, with on-call general practitioners (GPs), are the backbone of prehospital services [4, 5], and the majority of medical emergencies are handled by the ground ambulance staff, often in cooperation with the GPs.

The organization of prehospital services in Norway has changed over the last few decades in order to meet the requirements of new treatment algorithms and to fulfill the demands of health regulations. In the period of 2003–2013, acute missions with a ground ambulance increased almost 100% in Western Norway. On the other hand, use of HEMS was stable during the same period [6], though differences between HEMS bases have been reported [7].

Emergency Medical Communication Centers (EMCCs) provide medical advice and coordinate responses to medical emergencies. The HEMS is dispatched to patients with severe illness and/or trauma in need of specialized medical assessment, treatment, and/or rapid transport. Dispatch of the HEMS is not intended to replace on-call GPs [8]. However, changes in hospital organization and a focus on prehospital delay in the treatment of acute myocardial infarction, stroke, or trauma can potentially increase the use of HEMS in a rural country, such as Norway [9, 10].

From 2007 to 2016, the number of OOH casualty clinics in Norway decreased from 230 to 182 [11]. Driven by the need for organizational reinforcement [12], less duty time for the GPs, and more stable recruitment of personnel, several inter-municipal OOH districts were established. In the rural county of Sogn og Fjordane (S&F), nine municipalities were reorganized into one large inter-municipal OOH district in April 2009. The result was that one on-call GP was responsible for a larger geographic area with a greater number of inhabitants than they had been previously. Nationally, such reorganization into larger inter-municipal OOH districts has not resulted in better equipped OOH clinics, increased practical training, or more call-outs with a car from the OOH services. The competence of on-call GPs has been reported to be decreasing [13, 14]. Reports have also indicated an increase in HEMS missions, with clinical content handled previously by the on-call GPs. This development has been linked to major organizational changes in OOH services [15, 16]. Such organizational changes should be evaluated to determine whether they had the intended, or possibly adverse, effects. Therefore, we investigated whether HEMS dispatches increased in response to the organizational change when nine municipalities in S&F merged into one large inter-municipal OOH district on 1 April 2009.

## Methods

**Fig. 1:**
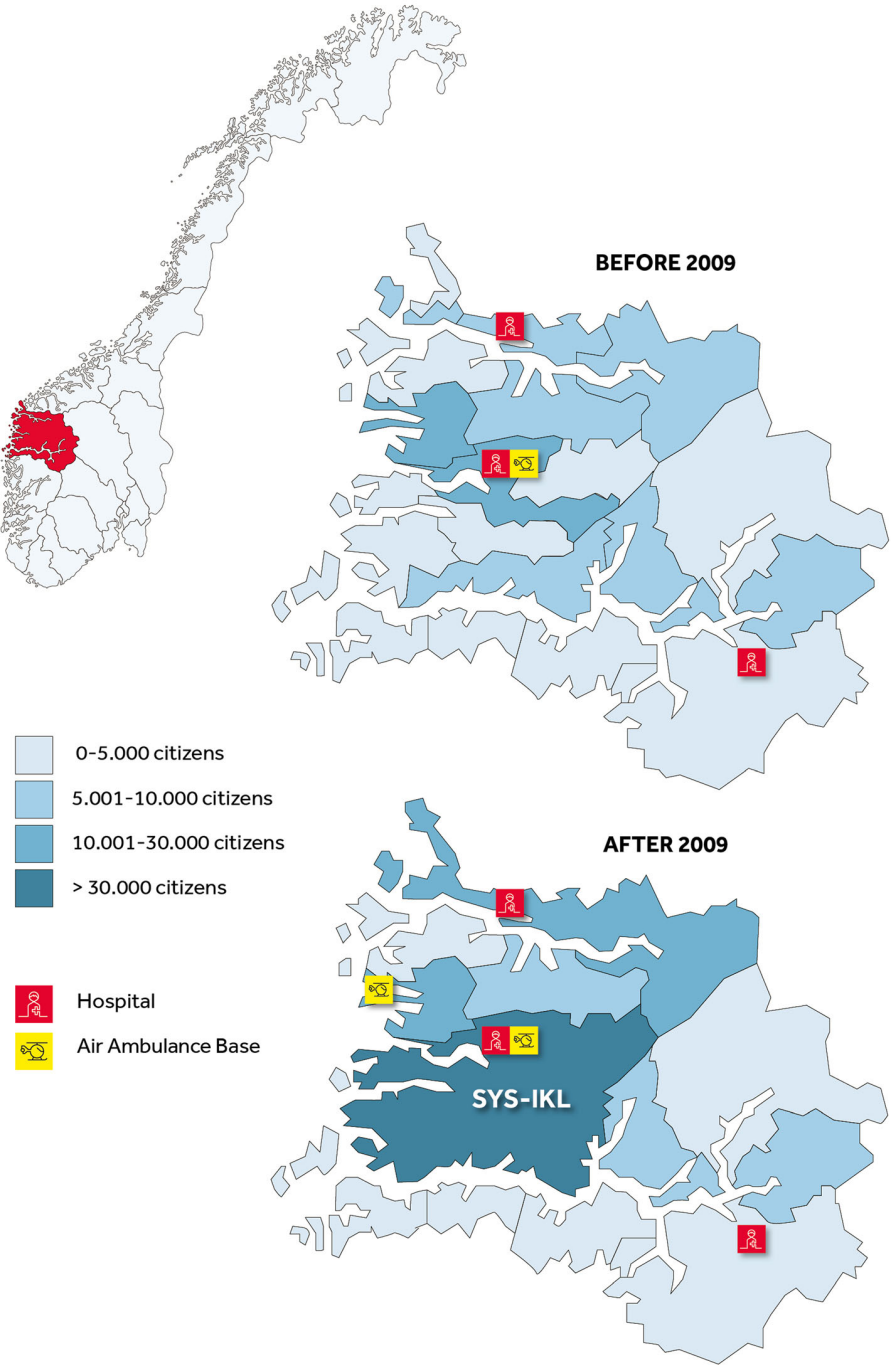
OOH-districts, air ambulance bases and hospital before and after 2009 in Sogn og Fjordane. SYS-IKL: A large inter-municipal casualty clinic covering nine municipalities in Sogn og Fjordane

S&F covers 18 623 km^2^ in Western Norway and is sparsely populated, with 110 000 inhabitants in 2019. One HEMS base is located in Førde, and one search and rescue (SAR) helicopter operates in Florø, a 45-min drive from Førde. Both the HEMS and SAR include an anesthesiologist in addition to a pilot and a rescue paramedic and operates 24/7/365 under challenging geography and weather condition. The HEMS base has a rapid response car available if weather conditions restrict flights (Fig. 1).

Each municipality in Norway is responsible for the OOH service, with at least one GP on-call and availability for call-outs in emergencies. On 1 April 2009, nine municipalities in S&F reorganized their OOH services, relocating all local OOH services to one large inter-municipal casualty clinic (SYS-IKL) in Førde, covering an area of 6 400 km^2^ and 35 000 inhabitants. As a result of this organizational change, the driving distance for patients can be up to 100 km. The median driving time for patients in the same area to SYS-IKL is 1 h 45 min [17]. Before 2009 the driving distance could be up to 45 km and 40 min driving time. Until January 2016, SYS-IKL had one doctor on-call at the clinic, and on weekends and holidays a doctor was on-call from home with the possibility to come to the casualty clinic upon request. The rest of S&F’s 17 municipalities had local (*n* = 8) or inter-municipal (*n* = 9) OOH services in 2009.

The change to SYS-IKL was a natural experiment with the possibility to evaluate use of the HEMS before and after the organizational change.

### Data

All HEMS dispatches in S&F from 1 January 2004 to 31 December 2013 were included. The Førde HEMS registers the patient data, timeline, and operational data, including the reason for cancellation, for all dispatches in their database “Airdoc”. Reasons for cancellation were: no longer medically indicated, bad weather conditions, concurrency conflicts, flight restrictions, or technical. Missions with a patient encounter also registered a severity score using the National Advisory Committee for Aeronautics (NACA) score [18]. Dispatches from other HEMS bases to missions in S&F were identified through the databases at the Ålesund, Bergen, Ål, and Dombås HEMS, Florø SAR Helicopter, and statistics from the National Air Ambulance Services. In addition, we compared the number of missions with the requests of HEMS from the EMCC in Førde.

Data were aggregated to obtain the total number of dispatches per month. SYS-IKL and MOS (municipalities outside SYS-IKL) subgroups were also created, and the municipalities categorized as urban or rural. Municipalities with > 7000 inhabitants were defined as urban. For comparison across subgroups, data were analyzed as dispatches per 1000 inhabitants. Distances between the municipalities and OOH service were measured using postal code coordinates [19] when available. The rest were measured between town hall in each municipality and OOH service.

### Statistical analysis

In order to evaluate the potentially significant effect of introducing the SYS-IKL organizational change on the number of dispatches, we applied interrupted time series regression (ITS) [20]. ITS is a regression model specifically developed for analyzing interventions introduced at a population level over a clearly defined time period when the pre-intervention and post-intervention period is clearly defined, has short-term outcomes, and has sequential measures with preferably equal numbers of data points distributed before and after the intervention [20].

The outcome variable in the analyses was the monthly aggregated number of HEMS dispatches. As this outcome variable is a count variable, our intended approach was to fit a Poisson ITS step change model. However, preliminary analyses indicated significant overdispersion, i.e., larger variation in the data than the Poisson model can handle. To adjust for this, we used a more general quasipoisson model. As HEMS dispatches are known to vary throughout the year [6], the model was further adjusted for seasonality using harmonic terms. In order to transform results to rates, population data were used as an offset variable in the regression model.

Differences in travel distance between the patients and on-call doctor for SYS-IKL and MOS were analyzed using Mann–Whitney-Wilcoxon non-parametric tests. Changes in NACA scores before and after the policy change were tested using chi-squared.

The statistical analyses were performed using SPSS Statistics Version 22/23 (IBM Corp., Armonk, NY, USA) ang R3.5.2 [21].

## Results

A total of 8751 HEMS dispatches were identified during the 10-year period. Of these, 5009 (57.2%) missions were completed with a patient encounter. The number of dispatches for individual years, both in total and for SYS-IKL and MOS, are presented in Table 1.

**Table 1:** HEMS dispatches in the Norwegian county of Sogn og Fjordane

Year	Total		SYS-IKLa		MOSb		Inhabitants
	Dispatches	Rate	Dispatches	Rate	Dispatches	Rate	
2004	695	0.54	197	0.49	498	0.56	107 222
2005	831	0.64	252	0.63	579	0.65	107 032
2006	896	0.70	246	0.62	650	0.74	106 650
2007	766	0.60	220	0.55	546	0.62	106 194
2008	834	0.65	257	0.65	577	0.66	106 259
2009	806	0.63	222	0.56	584	0.66	106 457
2010	989	0.76	243	0.61	746	0.84	107 080
2011	1012	0.78	254	0.63	758	0.85	107 742
2012	961	0.74	232	0.57	729	0.82	108 201
2013	961	0.74	244	0.60	717	0.80	108 700
All	8751		2367		6384		

Data are presented as total count and rates per 1000 inhabitants per month

^a^SYS-IKL: Nine municipalities in Sogn og Fjordane with one large inter-municipal casualty clinic

^b^MOS: Municipalities outside SYS-IKL in Sogn og Fjordane with both municipal and inter-municipal casualty clinics

**Fig. 2:**
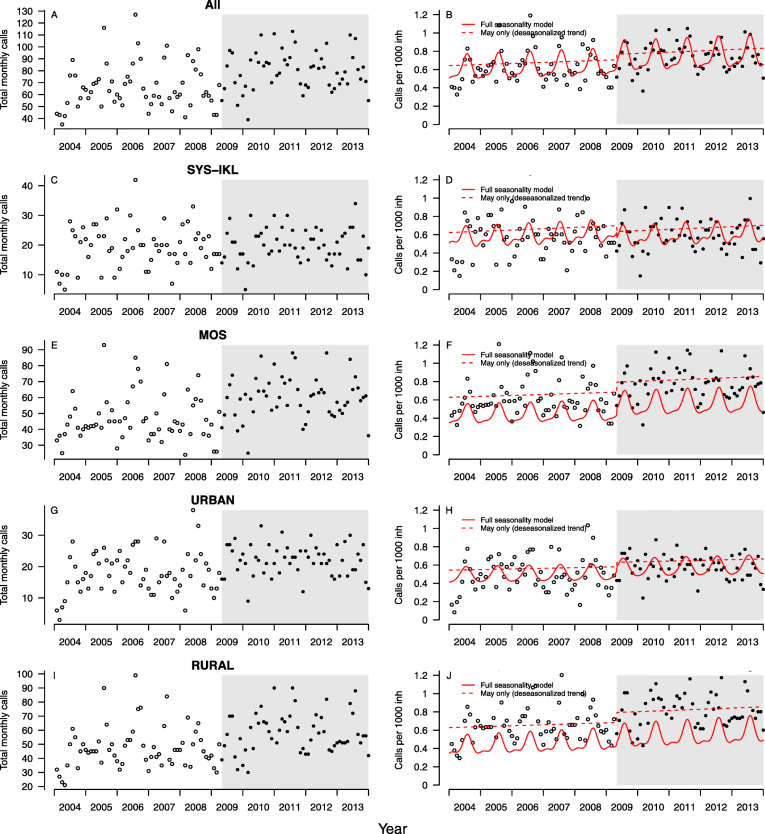
HEMS dispatches in Sogn og Fjordane, 2004–2013. Left, monthly total calls. Right, calls per 1000 inhabitants. The red dotted line indicates the fitted quasipoisson model with breakpoint, and the red solid line indicates further adjustment for seasonality. SYS-IKL: Nine municipalities in Sogn og Fjordane with one large inter-municipal casualty clinic. MOS: Municipalities outside SYS-IKL in Sogn og Fjordane with both municipal and inter-municipal casualty clinics

Total dispatches per month are shown in Fig. 2a. Fitting a quasipoisson regression model adjusted for seasonality (Fig. 2b), we found no significant change in HEMS dispatches after the policy change (*p* = 0.251).

Plotting the monthly number of dispatches for SYS-IKL (Fig. 2c) and MOS (Fig. 2e) separately, we found more dispatches for MOS. Adjusted per 1000 inhabitants, the monthly mean (SD) rate was 0.58 (0.22) and 0.60 (0.17) for SYS-IKL and 0.61 (0.19) and 0.78 (0.17) for MOS prior to and after the policy change, respectively. Fitting quasipoisson regression models and adjusting for seasonality (Fig. 2d and f respectively) resulted in no significant change for SYS-IKL (*p* = 0.400) and borderline significance for MOS (*p* = 0.056).

We found more HEMS dispatches in rural areas (Fig. 2 g and i). Fitting quasipoisson regression models adjusted for seasonality, we found a significant general linear increase in HEMS dispatches for the rural group over the whole time period (Fig. 2 h, *p* = 0.045), but no added increase after the policy-change (*p* = 0.502). The urban subgroup did not exhibit any significant changes over time in general (*p* = 0.506) or after introduction of the organizational change (Fig. 2j, p = 0.447).

Though there was generally no difference in the number of HEMS dispatches before and after the change in organization, travel distances to the OOH service increased significantly in SYS-IKL after the change, median [quartiles] (5.0[3.0, 6.2] km vs 26.5[5.0, 62.2] km, *p* < 0.001). Distance within MOS increased from 7.3 [5.1, 13.1] km to 8.3 [5.1, 22.0], *p* < 0.001. The distances were significantly higher for rural (8.9 [6.2, 22.0] km) than urban (5.0 [3.0, 8.3] km, *p* < 0.001).

NACA scores did not significantly change within the SYS-IKL, with a mean score of 3.99 before and 3.88 after the policy change (*p* = 0.070). Within MOS, the change was significant (*p* = 0.007), with a mean NACA score of 3.76 before and 3.84 after.

## Discussion

A change in HEMS dispatch rates between SYS-IKL and MOS after 1 April 2009 was not confirmed in our analysis. Though national guidelines exist for HEMS dispatch, the different bases in Norway use the service differently [22]. Rates of aborted or declined missions vary and depend on local routines. The EMCCs have varying HEMS dispatch criteria depending on available resources, demographics, and distance to hospitals with a capacity to handle trauma and other acute medical illnesses. A lower threshold for dispatching the HEMS can be a consequence in areas with long distances for patients to travel to health services. However, the stable dispatch of the HEMS in our analyses is similar to the findings in another recent study [6]. Our findings support that, despite an increase in emergency calls to the EMCC, increased ambulance missions and less frequently utilized the on-call doctor at the site, the HEMS still seemed to have the same threshold for dispatch.

Several studies have shown less frequent use of health services related to increased travel distance, such as lower rates of mammography screening [23], lower hospitalization rates for children [24], and major barriers to hospital referral by GPs [25]. Both decreasing use of OOH services and fewer medical situations categorized as acute due to longer distance between the patients and the OOH service are well-documented [26, 27]. We did not find any change in HEMS dispatch within SYS-IKL after 2009, despite the increased travel distance. However, over time, the rural municipalities had an increase in HEMS dispatch, but this applied to both municipalities with increased distance from the OOH service and municipalities with unchanged travel distance. Somehow, distance matters, but the correlation between HEMS dispatch and distance from OOH services is not consistent. A general centralization of many health services, including ground ambulance stations, OOH services, and hospitals, may contribute to the increase in HEMS dispatches. In 2012, only 0.5% of the contacts at the OOH service within SYS-IKL that resulted in a consultation with a doctor was responded with a call-out, and the annual report explained the low rate as consequences of having only one doctor on-call and the long distances to the patients [28]. The percentages of patients seen by both an on-call GP and HEMS decreased from 53% in 2006 to < 30% in 2015 in one area in Norway [22]. Some have described this as an abdication of the OOH services from large areas in Norway [29].

It seems plausible that the centralization of OOH services has resulted in an increased number of missions for the ground ambulances whereas this seems not to be the case for HEMS. Though the HEMS had a stable annual number of dispatches from 2004 to 2013 in the western part of Norway, acute missions with ground ambulances were increased by 95% in the same period [6]. In S&F, the total increase in ground ambulance missions was 27%, and nearly half of the increase occurred from 2009 to 2010. One explanation is that more patients require ambulance transport to the OOH services because the on-call doctor has a limited possibility of call-out. HEMS are only involved in a minority of the acute missions and are not affected by the structural change to the same degree as ground ambulances. Another reason for the increase in acute missions with ground ambulances can be the focus on triage using a decision tool called the Norwegian Index for Medical Emergencies [30]. This index is a criteria-based decision tool used in the EMCCs to classify the level of response. Though on-call doctors and the HEMS can accept or decline an acute mission when alarmed, ground ambulances are dispatched based on the level of response from the EMCC. A possible over-triage using the index and increased transport to OOH services, dispatch, and use of ground ambulance should be explored further, especially after structural changes in the OOH service.

Does it matter if ground ambulance workers, on-call GPs, or HEMS physicians encounter the patient? The main indication for dispatching the HEMS is a severe illness or trauma. Compared to transport and treatment by ground ambulances, the HEMS should have an anticipated gain in health outcomes and should not replace the on-call GP [8]. Concurrent missions can potentially be a problem if the HEMS is used as a compensatory mechanism due to unavailable on-call GPs. The crew can also have to decline missions if they have exceeded the duty hours. After working 14 h of the last 24 h, they are obliged to rest the next 8 h. These HEMS regulations help maintain flight safety [31]. As a consequence of the increased use of the HEMS, more missions could be declined despite of being medically warranted. On the other hand, equal access to health services regardless of residence is an important principle in Norwegian health policy. The HEMS can compensate for differences in the use of health services due to long distances.

Over-triage at the EMCC is both known and accepted to some extent [32]. However, on-call GPs play an important role as gatekeepers for reduced use of the health care system and to reduce hospitalizations [33]. The Norwegian health care system is based on the principal of using the lowest efficient level of care (LEON), and GPs cooperating with ambulance workers in acute missions reduce admissions to the hospital compared to ambulance workers alone [34]. In addition, GPs degrade the urgency/severity of many missions when on site [35]. On the other hand, a study from S&F looking at acute missions in which the HEMS had to cancel revealed no difference in treatment or reduced hospitalization when the on-call GP was on site or not [36]. Although improved patient care with on-call GPs on the site are demonstrated in some studies [34, 35], it is unclear which patients should be approached with a call-out reaction. Further studies are essential to offer recommendations on the capacity of OOH services and organizational matters.

The prehospital care system is organized differently in other countries, with limited involvement of the primary health care in acute medical situations [5]. However, equal access to health care in sparsely populated areas, resource allocations to HEMS, and dispatch criteria for HEMS, are highly relevant topics for discussion in many countries. After the establishment of a nationally organized HEMS in Finland in 2012, they discovered that a higher rate of requests were cancelled, compared to other countries. This could be due to over triage [37] and is an example of prehospital organizational changes which had an unintentional effect in a different area in the prehospital emergency service. Denmark uses anaesthesiologists in rapid response vehicles spread geographically around the country, in contrast to Norway’s use of GPs in the OOH services [38]. These two systems are expected to manage emergency patients and both have to rely on HEMS in some situations. Nevertheless, when the on-call OOH doctors disappeared from some geographical areas in Norway, HEMS utilization was not increased. Our findings contribute to the body of knowledge on how organizational changes can influence the use of HEMS, which is important to acknowledge prior to implementing new services and systems.

### Strengths and limitations

The present study has several strengths. First, all HEMS dispatches registered over 10 years were included. In addition, the ITS regression model is a well-established method for retrospective analysis of interventions introduced at a population level. However, the operational data used in our study were not registered with an intention to do research. Missing or wrong data is possible, especially considering that the data are registered during acute medical situations. We have checked different data sources (AirDoc, statistics from the Air Ambulance services and EMCC) with intentions to identify missing data and have not found a significant amount that influence the result. Furthermore, varying use of the HEMS between the bases in Norway is known [7] and should be taken into consideration if the results are compared to other areas. However, our analyses used rates before and after the organizational change within the area of one base and did not make a comparison between bases. To our knowledge, no other major system changes occurred in the study period. Theoretically, the establishment of a SAR base in 2009 could have had an impact on HEMS use. Still, the same dispatch criteria is used for requesting SAR, and SAR is usually requested when HEMS is unavailable due to concurrency, bad weather or technical reasons.

### Interpretations

Based on our findings, the increase in HEMS dispatches was less than expected due to the organizational change. In order to identify unintended consequences of changes in the prehospital services, such organizational changes should be evaluated more often with suitable methods. Our result indicate that use of HEMS is not significantly affected by centralization of OOH services, but changes in the use of ground ambulances should be evaluated in more detail.

## Conclusion

Reorganizing the local OOH services into one large inter-municipal OOH district did not result in an increase in HEMS dispatches. We found a trend, but not a statistically significant change, towards an increased use of HEMS in rural areas.

## Data Availability

The datasets used and analyzed during the current study are available from the corresponding author on reasonable request.

## Abbreviations

EMCC: Emergency Medical Communication Center
GP: General practitioner
HEMS: Helicopter Emergency Medical Service
ITS: Interrupted time series regression
MOS: Municipalities outside SYS-IKL
NACA: National Advisory Committee for Aeronautics
OOH: Primary care out-of-hours service
SAR: Search and rescue
S&F: County of Sogn og Fjordane
SYS-IKL: Large inter-municipal casualty clinic in Sunnfjord og Ytre Sogn
